# Dental-Derived Mesenchymal Stem Cells: State of the Art

**DOI:** 10.3389/fcell.2021.654559

**Published:** 2021-06-22

**Authors:** Bo Li, Takehito Ouchi, Yubin Cao, Zhihe Zhao, Yi Men

**Affiliations:** ^1^State Key Laboratory of Oral Diseases, West China School of Stomatology, Sichuan University, Chengdu, China; ^2^National Clinical Research Center for Oral Diseases, West China School of Stomatology, Sichuan University, Chengdu, China; ^3^Department of Orthodontics, West China School of Stomatology, Sichuan University, Chengdu, China; ^4^Department of Dentistry and Oral Surgery, School of Medicine, Keio University, Tokyo, Japan; ^5^Department of Physiology, Tokyo Dental College, Tokyo, Japan; ^6^Department of Head and Neck Oncology, West China School of Stomatology, Sichuan University, Chengdu, China

**Keywords:** mesenchymal stem cells, tooth regeneration, tissue engineering, bone defect reconstruction, immune modulation

## Abstract

Mesenchymal stem cells (MSCs) could be identified in mammalian teeth. Currently, dental-derived MSCs (DMSCs) has become a collective term for all the MSCs isolated from dental pulp, periodontal ligament, dental follicle, apical papilla, and even gingiva. These DMSCs possess similar multipotent potential as bone marrow-derived MSCs, including differentiation into cells that have the characteristics of odontoblasts, cementoblasts, osteoblasts, chondrocytes, myocytes, epithelial cells, neural cells, hepatocytes, and adipocytes. Besides, DMSCs also have powerful immunomodulatory functions, which enable them to orchestrate the surrounding immune microenvironment. These properties enable DMSCs to have a promising approach in injury repair, tissue regeneration, and treatment of various diseases. This review outlines the most recent advances in DMSCs’ functions and applications and enlightens how these advances are paving the path for DMSC-based therapies.

## Introduction

By revisiting history, the concept of mesenchymal stem cells (MSCs) and some relevant fundamental studies can be traced back to several decades ago (Bianco et al., 2008), which were associated with the breakthrough finding of an intrinsic osteogenic potential of bone marrow cells, as was revealed by heterotopic transplantation (Friedenstein et al., 1968). Subsequently, bone marrow stromal cells, a minor yet distinguishable subpopulation of bone marrow cells, were pointed out to possess osteogenic potential, as well as nonhematopoietic origin, rapid adherence to culture dishes, and colony-forming ability (Friedenstein et al., 1970). In 1991, the term MSCs was first coined by Caplan, proposing their multipotent differentiation ability to give rise to mesodermal lineage (Caplan, 1991). Thereafter, MSCs have been well known for their multipotency (Pittenger et al., 1999) and identified in a variety of tissues and organs since the formulation of the terminology, including, but not limited to, bone marrow, liver, heart, adipose tissue, and scalp tissue (Zuk et al., 2002; Shih et al., 2005; Beltrami et al., 2007).

**FIGURE 1:**
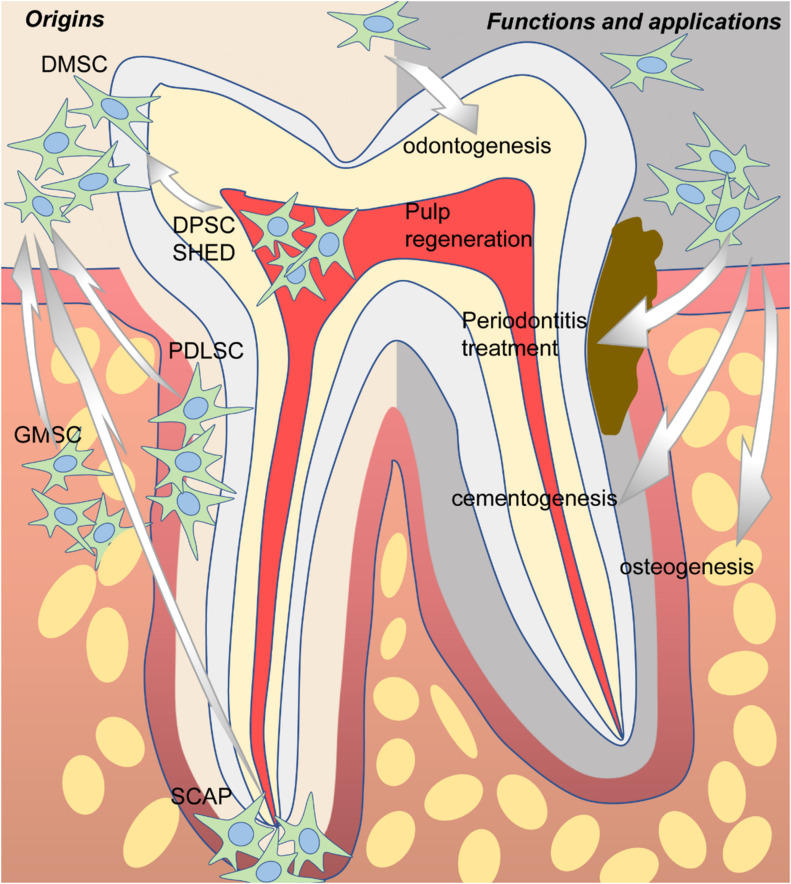
The origins, potential functions, and applications of dental-derived mesenchymal stem cells (DMSCs).

In 2000, a clonogenic population of dental pulp cells was first isolated by Gronthos et al. (2000) and identified as MSCs due to similar properties as bone marrow stromal cells (BMSCs) in terms of immunoreactivity profile, as well as the capacity for self-renewal and the potential for multidirectional differentiation. Since then, more and more dental-derived cells have been found to possess stem cell properties and were named according to their tissue of origin, including dental pulp stem cells (DPSCs) (Gronthos et al., 2000), stem cells from human exfoliated deciduous teeth (SHEDs) (Miura et al., 2003), periodontal ligament stem cells (PDLSCs) (Seo et al., 2004), dental follicle stem cells (DFSCs) (Handa et al., 2002; Morsczeck et al., 2005), stem cells from apical papilla (SCAPs) (Sonoyama et al., 2006, 2008), and gingival mesenchymal stem cells (GMSCs) (Zhang et al., 2009) (Figure 1). Overall, all the abovementioned dental-derived stem cell populations can be termed collectively as dental-derived mesenchymal stem cells (DMSCs) (Sharpe, 2016). In this review, we will focus on introducing and discussing the most up-to-date advances of DMSCs concerning functions, applications, and beyond.

## Differentiation and Function

### Dentinogenesis

Odontoblasts are extremely specialized cells involved in the deposition and mineralization of the dentin matrix, namely, dentinogenesis or dentin formation. The functions of odontoblasts include the formation and regeneration of the dentin–pulp complex (Kawashima and Okiji, 2016). DPSCs and SHEDs are two subpopulations of DMSCs that perform as major resources of odontoblasts during tertiary dentin formation upon postnatal injury since the preexisting odontoblast cannot produce reparative dentin (Tatullo et al., 2015; Mortada and Mortada, 2018). The standard procedure for odontogenic differentiation can be induced *in vitro* by culturing DMSCs in odontogenic medium containing dexamethasone, β-glycerophosphate, and ascorbic acid (Langenbach and Handschel, 2013). Under such culture conditions, DMSCs subsequently express osteoblast-associated markers, including alkaline phosphatase (ALP), collagen type 1 (COL1), osteopontin (OPN), osteocalcin (OCN), dentin matrix acid phosphoprotein 1 (DMP1), matrix extracellular phosphoglycoprotein (MEPE), and dentin sialophosphoprotein (DSPP). These markers are equally widely used as odontoblasts-specific differentiation markers in plenty of studies (Dong et al., 2019; Iezzi et al., 2019). Besides the abovementioned markers, previously unrecognized odontoblast markers have been identified, such as NOTUM and SALL1 (Krivanek et al., 2020), by single-cell RNA sequencing (scRNA-seq), which is a seminal technique developed in recent years and enables researchers to explore transcriptional profiles at the level of a single cell with unprecedented resolution. Interestingly, scRNA-seq uncovered novel functions of some well-defined odontoblast markers as well, for instance, the role of DSPP in amelogenesis (Chiba et al., 2020).

To date, researchers have explored different induction conditions, which aim to promote or impair odontogenic differentiation. Allogeneic fibrin clot (AFC) serum exhibited sufficient cytokines as well as growth factors to induce odontogenic differentiation of human DPSCs and human PDLSCs *in vitro* (Cao et al., 2020). *Sapindus mukorossi* seed oil has also been proven to increase the ALP activity and the secretion of mineralized nodules of DPSCs, and can promote osteogenic or odontogenic differentiation and matrix vesicle secretion of DPSCs under odontogenic induction (Shiu et al., 2020). In contrast, alcohol can suppress DPSC odontogenic differentiation and mineralization, by decreasing the ALP activity, attenuating the formation of mineralized nodules, and suppressing the expression of odontoblastic markers, including ALP, DSPP, DMP1, and OCN. Mechanistically, alcohol negatively regulates odontogenic differentiation through the mechanistic target of rapamycin (mTOR) signaling in a dose-dependent manner (Qin et al., 2017). Hence, quite a few elements are associated with the process of odontogenic differentiation.

Also, some other signaling molecules participate in the odontogenic differentiation process. Copine 7 (CPNE7) works as a diffusible signaling molecule and was proven to induce non-dental MSCs to differentiate into odontoblasts *in vitro via* the control of DSPP expression (Oh et al., 2015). Also, CPNE7 has been demonstrated to promote the formation of dentin-like tissues *in vivo* (Oh et al., 2015). A similar pro-odontogenic effect was found in endothelin-1 (ET-1), which can promote the odontoblastic differentiation of DPSCs (Liu M. et al., 2018). Receptor tyrosine kinases (RTKs) played a pivotal role in cell fate decisions, of which ephrin receptors (Eph) make up the largest known subgroup. After odontogenic induction, EphrinB2 (ligand) and its cognate receptors EphB2 and EphB4 were upregulated in DPSCs in both gene and protein levels, and meanwhile, EphrinB2 signaling showed enhanced effects on osteogenic/odontogenic differentiation of human DPSCs (Heng et al., 2018). Other regulatory molecules are lysine demethylase 6B (KDM6B) (Xu et al., 2013) and pentraxin-3 (PTX3) (Kim Y. et al., 2019). Mechanistically, KDM6B was recruited to BMP2 promoters, leading to the removal of epigenetic marks H3K27me3, activation of *BMP2* transcription, and thus, odontogenic differentiation of DMSCs (Xu et al., 2013). As for PTX3, it was indicated to be positively correlated with osteogenic or odontogenic differentiation of human DPSCs (Kim Y. et al., 2019). The relationship and interaction between different molecules are still unraveled, and it remains to be further explored and elucidated for the underlying mechanisms of odontogenic differentiation.

Apart from culture conditions and signaling molecules, RNA was demonstrated to have an impact on the process of odontogenic differentiation. For instance, miR-223-3p knockdown was proven to increase SMAD3 and inhibit odontogenic differentiation (Huang X. et al., 2019). Upregulated miR-21 was revealed to associate with increased odontogenic differentiation of DPSCs in a tumor necrosis factor-α (TNF-α)-mediated manner (Xu et al., 2018). Meanwhile, miR-143-5p and hsa-let-7c showed the inhibition of odontogenic differentiation of DMSCs (Ma et al., 2016; Liu G.X. et al., 2018; Zhan et al., 2018). Long non-coding RNAs (lncRNAs) have been reported to involve in dentin development and facilitate the odontogenic differentiation of human DPSCs (Zeng et al., 2018). To conclude, odontogenic differentiation and dentinogenesis are governed by a variety of signaling molecules, growth factors, miRNAs, and specific culturing conditions, resulting in a complex and intertwined regulatory network.

### Cementogenesis

Cementogenesis refers to the process of cementum formation, which covers the roots of teeth by cementoblasts. Cementoblasts, whose biological function is cementogenesis, are the key cell type responsible for anchoring the periodontal ligament to the tooth (Zhao et al., 2021). In general, cementoblasts can be found within periodontal tissue and play a crucial role in periodontal complex stabilization and regeneration. Furthermore, it has been validated that DMSCs, especially PDLSCs and DFSCs, can differentiate into cementoblasts (Hyun et al., 2017; Zhai et al., 2019; Yang J.W. et al., 2020).

Similarly, extensive studies have been conducted to better understand cementogenic differentiation concerning RNA interference and signaling pathways. Ets variant 1 (ETV1), a differentiation-related transcription factor that reciprocally regulates miR-628-5p and miR-383 coordinately in a feedback loop, is thought to be necessary for cementogenesis (Iwata et al., 2021). Another study focused on miR-361-3p, which decreased during cementoblastic differentiation, while inhibition of miR-361-3p resulted in increased cementoblastic differentiation (Liao et al., 2019). Moreover, the Wnt/β-catenin (WNT) pathway was blocked by forced expression of miR-361-3p in cementoblastic differentiation, whereas multiple other pathways are notably activated, including the extracellular signal-regulated kinase 1/2 (ERK1/2), c-Jun N-terminal kinase (JNK), p38 mitogen-activated protein kinase (p38/MAPK), phosphoinositide 3-kinase (PI3K) protein kinase B (AKT) (PI3K/AKT), and nuclear factor kappa-light-chain-enhancer of activated B-cells (NF-κB) pathways (Liao et al., 2019).

As for signaling pathways, canonical WNT signaling might positively regulate the expression of cementogenic-related markers of DFSCs, including runt-related transcription factor 2 (RUNX2), ALP, OCN, cementum attachment protein (CAP), and cementum protein (CEMP) (Du et al., 2012; Nemoto et al., 2016). A potential link between WNT signaling and the plasminogen-plasmin pathway to control cementogenic differentiation has been reported (Martins et al., 2020). Besides, the PI3K/AKT and JNK pathways have also been identified to participate in the enhanced cementogenesis of PDLSCs, which can be activated by M2 macrophages through cytokine and/or chemokine secretion, in particular, interleukin-10 (IL-10) and vascular endothelial growth factor (VEGF) (Li et al., 2019).

However, the characterization of DMSCs committed to undergo osteoblast/cementoblast differentiation remains largely unknown (Saito et al., 2014). Some proteins, which have been demonstrated to play vital roles in other lineage commitment and differentiation, can influence the cementoblast lineage cells as well. For instance, Sirtuin 6 (SIRT6) is essential for osteogenic differentiation, but it also inhibits glucose transporter 1 (GLUT1), a glucose transporter necessary in cementogenesis, and inhibited cementoblast differentiation by activating the AMP-activated protein kinase (AMPK) pathway (Huang L. et al., 2019). Furthermore, another good example is amelogenins. Amelogenins are crucial components of developing extracellular enamel matrix, which direct the organization and mineralization of enamel. Studies have demonstrated that C-terminus of amelogenins induces division, differentiation, and maturation of MSCs, especially in cementoblastic lineage cells (Kunimatsu et al., 2017, 2018). Additionally, cementoblastic differentiation of mesenchymal progenitor cells is tightly maintained by an autocrine system mediated by parathyroid hormone (PTH)-related peptide (PTHrP) and the PTH/PTHrP receptor, thereby preventing DMSCs from premature differentiation into cementoblasts (Takahashi et al., 2019). However, there is still insufficient research investigating this aspect, which requires more studies before clinical applications.

### Osteogenesis

**FIGURE 2:**
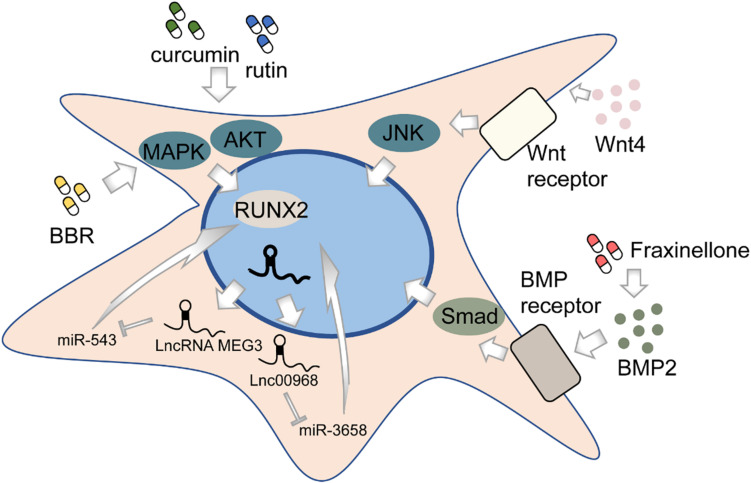
The regulation of osteogenic differentiation of DMSCs.

Osteogenesis, in brief, is the process of bone formation by osteoblasts. Among all subpopulations of DMSCs, DPSCs, and PDLSCs are most extensively studied. DPSCs cultured *in vitro* can differentiate into osteoblast-like cells in addition to its well-known potential to differentiate into odontoblasts (Mortada and Mortada, 2018) (Figure 2). This process is deemed to be regulated by various pathways, though not completely elucidated. The BMP-4/Smad signaling pathway is essential for the osteogenic differentiation of DPSCs, which can be suppressed by tumor necrosis factor-inducible protein-6 (TSG-6) (Wang Y. et al., 2020). The impaired osteogenic capacity of DPSCs in the inflammatory microenvironment can be rescued by WNT4 overexpression, which subsequently may function by affecting JNK signaling pathways (Zhong et al., 2019, 2020). LncRNAs, emerging as novel regulators, might play an important role in the osteogenesis of DPSCs by regulating gene expression. Transcriptome microarray identified a series of differentially expressed lncRNAs during osteogenic differentiation in human DPSCs (Zhang et al., 2017). Meanwhile, different lncRNAs may affect the expression of the same target oppositely, for example, RUNX2. To illustrate, lncRNA MEG3 inhibited human DPSCs osteogenic differentiation and promoted RUNX2 degradation via miR-543/SMURF1/RUNX2 regulatory network (Zhao et al., 2020c), whereas lncRNA LINC00968 promoted osteogenic differentiation and bone formation *in vitro* and *in vivo* and upregulated RUNX2 expression through competitive binding of miR-3658 (Liao et al., 2020). Besides, some chemical compounds, such as berberine (BBR), were recently identified to contribute to the osteogenic differentiation of DPSCs (Xin et al., 2020).

Like DPSCs, PDLSCs can also differentiate into osteoblast-like cells and highly expressed osteogenesis-related markers (Seo et al., 2004). Likewise, lncRNAs are associated with the osteogenic differentiation process of PDLSCs (Qu et al., 2016). Depleting lncRNA ANCR in progenitor-containing cell populations led to rapid differentiation gene induction (Kretz et al., 2012). A recent study shows that the silencing of a novel circular RNA (circRNA), circCDK8, can promote osteogenic differentiation of PDLSCs *via* repressing endoplasmic reticulum (ER) stress, autophagy, and apoptosis in a hypoxic microenvironment (Zheng et al., 2021). Furthermore, the effects of many chemical compounds on PDLSCs have been explored. Curcumin, a natural phenolic product derived from the turmeric rhizome, has been indicated to have a variety of biological functions including anti-inflammation, anti-cancer, and antioxidation (Aggarwal et al., 2007; Anand et al., 2007). Recent works have shown that curcumin may be a promising substance to promote the osteogenic differentiation in human PDLSCs by increasing early growth response protein 1 (EGR1) expression (Shi et al., 2021) and activating PI3K/AKT/Nrf2 signaling pathway (Xiong et al., 2020). Fraxinellone, a programmed death-ligand 1 (PD-L1) inhibitor, could alleviate inflammation and strengthen osteogenic differentiation of lipopolysaccharide (LPS)-stimulated PDLSCs through BMP2/Smad pathway (Fu et al., 2021), suggesting the possibility of fraxinellone in bone tissue regeneration. Rutin, a bioflavonoid distributed in fruits and vegetables, was found to significantly enhance the proliferation and osteogenic differentiation of PDLSCs through PI3K/AKT/mTOR signal pathway (Zhao et al., 2020a). In addition, both rutin and another herbal extraction, resveratrol, can protect human PDLSCs from TNF-α-induced damage to osteogenesis (Yuan et al., 2020; Zhao et al., 2020b). The recombinant chimeric protein of angiopoietin-1 (ANG-1) and a short domain of cartilage oligomeric matrix protein (COMP) could also exert the osteogenic role via PI3K/AKT pathway (Kook et al., 2014). Besides biological or chemical factors, physical stimulus, such as laser irradiation, may affect bone formation as well (Abramovitch-Gottlib et al., 2005; Kushibiki et al., 2015). However, the evidence seems inadequate to draw a conclusion, as no difference is observed between positive control and laser-irradiated groups (Pinheiro et al., 2018; Gholami et al., 2020). Further pertinent studies are needed to validate the efficacy of physical interventions on osteogenesis.

Although studies on DPSCs and PDLSCs are accumulated the most, other subpopulations of DMSCs have also been tested for their osteogenic potential. Both DFSCs and SHEDs possess osteogenesis capacity, while SHEDs are considered prospective seeding cells for use in stem cell-mediated bioengineered tooth root regeneration because of their easy accessibility (Yang X. et al., 2019). SCAPs have osteogenic potential as well and can be enhanced by secreted frizzled-related protein 2 (SFRP2), an antagonist of the canonical WNT signaling pathway (Yu et al., 2016). Last but not the least, transplantation of GMSCs effectively contributed to bone defect regeneration, which suggests their strong osteogenic potential (Tomar et al., 2010; Wang et al., 2011; Yang et al., 2013).

### Chondrogenesis

Chondrogenesis, namely, the formation of cartilage, is directed by chondrocytes. MSCs have chondrogenic potential, which is usually clarified by culturing in the chondrogenic medium as a micromass (Ichinose et al., 2005) and evaluated by Alcian blue staining. The expression level of chondrogenic markers, for instance, collagen II (COL2), collagen V (COL5), and sex-determining region Y box protein 9 (SOX9) can further confirm the differentiation status. Previously, primary synovial MSCs were proof of chondrogenic ability (Kohno et al., 2017). As expected, DMSCs were also validated to have the chondrogenic capacity (Huang et al., 2009). It has been reported that inhibition of ERK1/2 inhibited chondrogenic differentiation of DPSCs (Ba et al., 2017), whereas contradictory findings were shown previously in another paper (Dai et al., 2012), which suggests a complex regulatory mechanism underlying chondrogenic differentiation process. Distal-less homeobox 5 (DLX5) and homeobox C8 (HOXC8) enhanced the chondrogenic differentiation of SCAPs, and their overexpression upregulated the transcriptional activity of COL2, COL5, and SOX9 (Yang H. et al., 2020). Fas cell surface death receptor ligand (FasL) stimulation brought chondrogenic differentiation of human PDLSCs to a higher level, with the evidence of more collagen deposition and the presence of acid mucins and glycosaminoglycans (Pisciotta et al., 2020). Epigenetic modifications also play a role in regulating chondrogenic differentiation. For example, the upregulation of KDM6A could promote chondrogenesis of PDLSCs by demethylation of H3K27me3 (Wang et al., 2018). Interestingly, an extract of *Enterococcus faecium* (*E. faecium*), called L-15, has been reported to improve chondrogenic differentiation of human DPSCs (Kim H. et al., 2019). Briefly, *E. faecium* is a commensal in the gastrointestinal tract, and researchers found that its L-15 extract promoted the expression of early-stage chondrogenic markers, including SOX9, collagen type II alpha 1 (COL2A1), and aggrecan (ACAN), and suggested that L-15 at a concentration of 50 μg/ml was safe for *in vitro* chondrogenic induction (Kim H. et al., 2019).

### Myogenesis

The process of generating muscle or the formation of muscular tissue is recognized as myogenesis. Due to the potential versatility and easy accessibility, DMSCs are considered to be applicable as precursors for myogenic differentiation. Several studies have demonstrated that DPSCs possess the capacity of differentiating into smooth muscle cells (SMCs) *in vitro* under myogenic induction (Song et al., 2016; Jiang et al., 2019; Ha et al., 2021). Most of the studies focus on bladder SMCs as well as vascular SMCs (Song et al., 2016). The mechanism of the myogenic differentiation process of DPSCs remains unclear. The canonical WNT signaling pathway is well known to play an essential role in cell fate determination. Indeed, WNT signaling is involved in the process of DPSC differentiation into SMCs, in combination with multiple necessary growth factors, including transforming growth factor-beta (TGF-β1), basic fibroblast growth factor (bFGF, also known as FGF2), epidermal growth factor (EGF), hepatocyte growth factor (HGF), platelet-derived growth factor (PDGF), and VEGF (Jiang et al., 2019). To be noted, TGF-β1 may be one of the most important supplements to induce DMSCs to differentiate into SMCs. Mechanistically, TGF-β1 positively controls the SMC differentiation process of periodontal ligament-derived cells, possibly at the early stage of the lineage commitment, via Smad- and p38/MAPK-dependent manners (Yoshida et al., 2012). In contrast, fibroblast growth factor 1 (FGF1, also known as aFGF) suppressed the SMC differentiation of periodontal ligament-derived cells via ERK1/2 signaling (Takahashi et al., 2012).

### Angiogenesis

**FIGURE 3:**
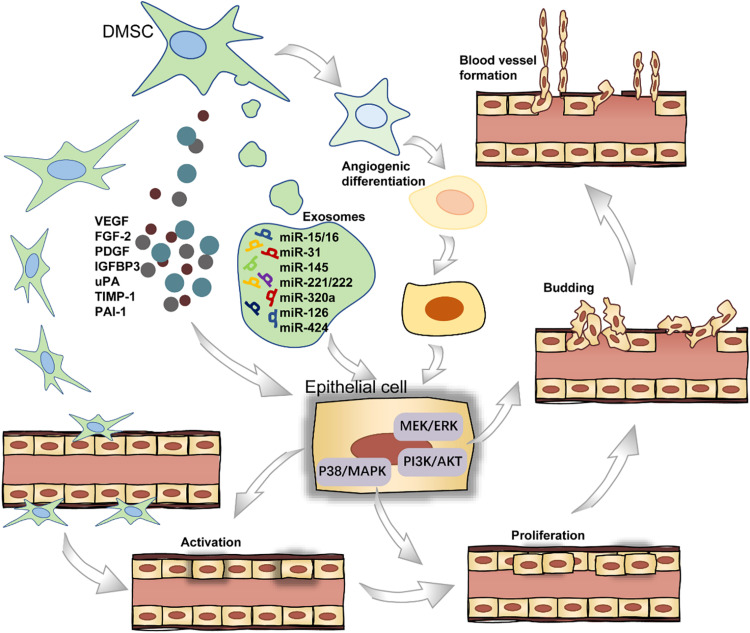
The role of DMSCs in blood vessel formation.

Angiogenesis refers to the establishment of new blood vessels from the preexisting vasculatures, occurs throughout life, and consists of four stages (Figure 3). In short, the endothelial cells (ECs) receive pro-angiogenic signals (stage I), followed by vascular fenestration (stage II), then partial endothelial to mesenchymal transition (EndoMT) (stage III), and in the end, new blood vessels sprouting (stage IV) (Zimta et al., 2019). DMSCs can secrete a wide range of pro-angiogenic factors including VEGF, FGF2, and PDGF in stage I, which bind to their corresponding receptors on ECs (Tonnesen et al., 2000; Tran-Hung et al., 2008). Furthermore, DPSCs can express monocyte chemoattractant protein-1 (MCP-1), also known as C–C motif chemokine ligand 2 (CCL2), to activate the ECs and stimulate ECs migration in the local microenvironment (Bronckaers et al., 2013). DPSCs, when co-cultured with the ECs, could stimulate EC proliferation through the p38/MAPK pathway and the secretion of FGF2 and VEGF in stage II (Tran-Hung et al., 2006). In stage III, the activation of the PI3K/AKT and MEK/ERK signaling pathways stimulates ECs residing in the interior layer of a blood vessel (Song and Finley, 2020). In a recent study, the gene-modified DPSCs, transfected with VEGF or stromal cell-derived factor 1α (SDF-1α) using lentiviral particles, were more capable of proliferation and stimulating the sprouting of the capillary tube-like structures in stage IV (Zhu et al., 2019). For a successful induction of local neoangiogenesis, two major cell populations must be taken into account: stem cells and vascular ECs. The cellular communication between these two types of cells could be mediated by exosomes. Especially, these extracellular vesicles are loaded with microRNA species, including miR-15/16, miR-31, miR-145, miR-221/222, miR-320a, miR-126, and miR-424 in angiogenesis-related progress (Gonzalez-King et al., 2017).

Dental-derived mesenchymal stem cells not only stimulate blood vessel formation via paracrine angiogenic factors but also participate directly in angiogenesis by differentiating into ECs (d’Aquino et al., 2007). As for the endothelial differentiation potential of DMSCs, DPSCs are able to differentiate into endothelial-like cells in the presence of VEGF. Previous work showed successful endothelial induction of DPSCs under a certain cell plating concentration when cultured in a specialized VEGF-added and serum-free culture medium (Karbanová et al., 2011). Also, DPSCs can functionally resemble perivascular supporting cells and induce more blood vessels when directly co-cultured with ECs (Janebodin et al., 2013; Saghiri and Asatourian, 2015). Intriguingly, DPSCs can also produce anti-angiogenic factors, including endostatin, insulin-like growth factor-binding protein 3 (IGFBP3), urokinase-type plasminogen activator (uPA), tissue inhibitor matrix metalloproteinase 1 (TIMP-1), and plasminogen activator inhibitor 1 (PAI-1) (Bronckaers et al., 2013). Collectively, these studies indicate that DMSCs could be potentially applied in the vascularization of regenerated dental tissues.

### Neurogenesis

**FIGURE 4:**
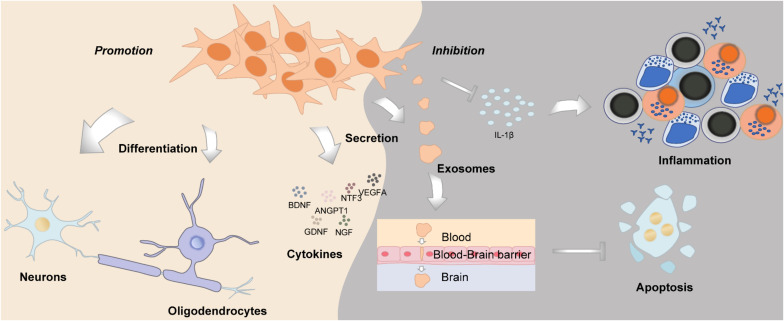
The role of DMSCs in neurogenesis and neuroregeneration.

Neurogenesis is defined as a process of generating functional new neurons from neural stem cells and precursors; DMSCs are cranial neural crest derived, with huge potential in neural repair and nerve regeneration, which could function similarly as Schwann cells (Pisciotta et al., 2013) (Figure 4). DMSCs possess the capacity for both neuronal and glial differentiation. In fact, it has been demonstrated that DMSCs express specific neural markers like nestin, glial fibrillary acidic protein (GFAP), β-III-tubulin, synaptophysin, and S100 protein (Sonoyama et al., 2008; Martens et al., 2012). Furthermore, the percentage of cells expressing neural markers in DMSCs is relatively higher than BMSCs. It has been reported that human DPSCs express significantly higher levels of neurotrophins and elicit more extensive innervation than human BMSCs (Pagella et al., 2020). DMSCs, in particular DFSCs, could differentiate into mature neurons and oligodendrocytes following spinal cord injury (Yang et al., 2017). Under neurogenic induction, DPSCs could also form a stellate neuron-like phenotype, which resembled the characteristics of functional neurons (Wang et al., 2019). Besides morphology, DPSCs also exhibited the capacity to generate a sodium current when exposed to a neurogenic medium, which was functionally consistent with neuronal cells (Arthur et al., 2008). Additionally, brain-derived neurotrophic factor (BDNF) and neurotrophin-3 (NT-3) can reprogram human DPSCs to neurogenic and gliogenic neural crest progenitors. Human DPSCs cultured in a serum-free medium with BDNF and NT-3 exhibited a greater potential to differentiate toward neuronal and Schwann glial lineage cells (Luzuriaga et al., 2019). Furthermore, DPSCs and SHEDs treated with BDNF, NT-3, and glial cell line-derived neurotrophic factor (GDNF) could differentiate into spiral ganglion neuron-like cells, which express neuron-specific β-III-tubulin, GATA-binding protein 3 (GATA3), and tropomyosin receptor kinase B (TrkB), protein markers of spiral ganglion neurons (Gonmanee et al., 2018). As for the aspect of function, intracellular calcium dynamics could be observed, which reflected neurotransmitter release (Gonmanee et al., 2018, 2020). DPSCs transfected with the human *Olig2* gene, an essential transcription factor for lineage determination of oligodendrocytes, would differentiate into oligodendrocyte progenitors (Askari et al., 2015). Taken together, DMSCs have their inherent advantages in neural repair and neurogenesis, such as they derived from the cranial neural crest, as well as their neurotrophic and neuroprotective properties, which suggest that DMSCs may be an ideal cell source for neural tissue regeneration.

### Hepatogenesis

The process of giving rise to or forming liver tissue is known as hepatogenesis, which also includes the structural and functional development of the liver. Over a decade ago, the expression of the liver-specific gene *Cyp7a1* revealed hepatogenic differentiation in embryoid body-derived stem cells (Asahina et al., 2004, 2008). In more recent years, a research group reported that SHEDs supplemented with hepatic growth factors, including dexamethasone, insulin–transferrin–selenium-X (ITS-X), and oncostatin, were proven to have quite a few cells positive for specific hepatic markers including α-fetoprotein (AFP), albumin, and hepatic nuclear factor 4α (HNF-4α) after induction (Ishkitiev et al., 2010). To be noted, the hepatogenic-differentiated SHEDs could store glycogen and produce urea, which suggested the cells commenced to function as hepatocytes (Ishkitiev et al., 2010). Later on, the same group demonstrated that both SHEDs and DPSCs could differentiate into high-purity hepatocyte-like cells in a serum-free medium, which might serve as a novel source for hepatic lineage differentiation for transplantation in the future (Ishkitiev et al., 2012). Melatonin could promote hepatic differentiation of human DPSCs by modulating various signaling pathways (Cho et al., 2015). Actually, not only DPSCs but also DFSCs, SCAPs, and BMSCs all had hepatogenic potential. Among these MSCs, DPSCs reflected the best hepatogenic potential compared with the other three cell types (Kumar et al., 2017). More importantly, recent studies from different groups confirmed that cryopreserved human DPSCs could differentiate into functional hepatocyte-like cells (Chen et al., 2016; Han et al., 2017), which implicated the promising potential of a personal DMSC banking as a possible source of tailor-made hepatocytes in the future (Ohkoshi et al., 2018).

### Adipogenesis

Adipogenesis is the formation of adipocytes, fat cells, from precursor stem cells. Currently, published data are conflicting about the potential and the time required for DMSCs to achieve adipogenic differentiation *in vitro*. During the adipogenesis of DMSCs, related components of the peroxisome proliferator-activated receptor (PPAR) signaling pathway, including PPAR gamma (PPAR-γ, also known as PPARG), fatty acid-binding protein 4 (FABP4), and the CCAAT/enhancer-binding protein (C/EBP) family were significantly upregulated (Nozaki and Ohura, 2011). Although some studies have reported that DMSCs can achieve adipogenic differentiation as efficiently as other MSCs, sometimes their conclusions were based solely on microscopic images of a few differentiated cells without further confirmation (Gronthos et al., 2000; Isobe et al., 2016). Meanwhile, DPSCs cultured in the adipogenic induction medium did not show cytoplasmic lipid accumulation and had low expression levels of adipogenic-related genes (Fracaro et al., 2020). A recent study disclosed the gene expression profile of DMSCs, which supported their limited capacity of differentiating into adipocytes (Fracaro et al., 2020). However, it seems that the adipogenic limitation is not insurmountable. As is revealed in another study, PIN1, a peptidyl-prolyl *cis/trans* isomerase, was identified to serve as a key regulator during adipogenesis of DMSCs. The overexpression of PIN1 via adenoviral infection in human DPSCs increased adipogenic differentiation while inhibiting odontogenic differentiation (Lee et al., 2014). In summary, we may have to admit that DMSCs do have difficulties in adipogenesis, but undoubtedly, DMSCs still possess adipogenic differentiation potential.

### Immunomodulation

**FIGURE 5:**
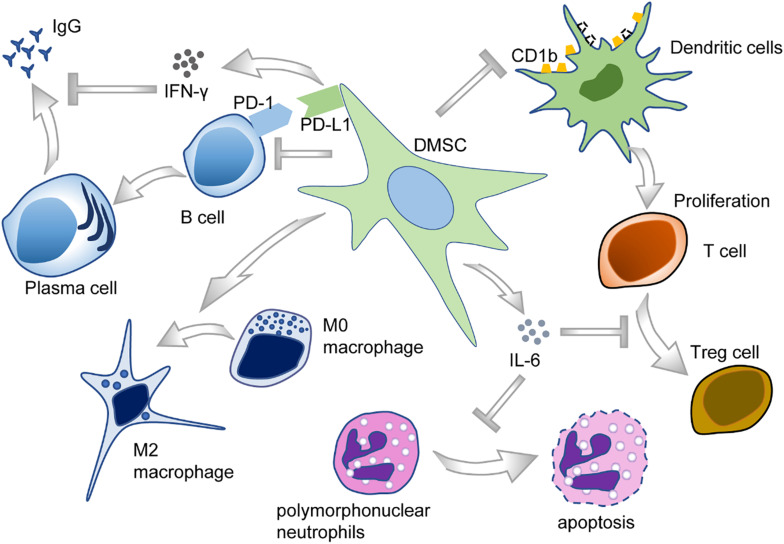
The immunomodulatory functions of DMSCs.

The oral cavity is the habitat of various microorganisms, and host–microbe homeostasis is an important aspect in the maintenance of oral health. Oral diseases are usually related to the imbalance of microbial flora and the invasion of microorganisms into oral tissues, sometimes even leading to systemic diseases. DMSCs may participate in immunomodulation by interacting with both innate and adaptive immune cells in their microenvironment (Andrukhov et al., 2019; Wang M. et al., 2020) (Figure 5).

Periodontal ligament stem cells, isolated from periodontal ligament tissues, exert a unique immunomodulatory property (Yu et al., 2019) and are capable of modulating a variety of immune cells. To illustrate, PDLSCs could suppress T-cell proliferation and disrupt T-cell differentiation into regulatory T-cells (Tregs) after being treated with D-mannose (Guo et al., 2018). PDLSCs also cause T-cell proliferation disorders through downregulating the expression of non-classical major histocompatibility complex (MHC)-like glycoprotein CD1b on dendritic cells (DCs) (Shin et al., 2017). Furthermore, PDLSCs also reduce the apoptosis of polymorphonuclear neutrophils (PMNs), the most abundant circulating blood leukocytes, through IL-6 secretion (Wang Q. et al., 2017). Moreover, PDLSCs inhibited B-cell activation through cell–cell interaction, which is primarily mediated by PD-1 and PD-L1 (Liu et al., 2013). As for macrophages, PDLSCs could potentiate the polarization toward M2 macrophages by stimulating arginase-1 (ARG1), CD163, and IL-10 and inhibiting TNF-α (Liu et al., 2019). Plus, PDLSCs could suppress the expression of TNF-α by macrophages, contributing to periodontal tissue regeneration (Nagata et al., 2017).

Besides PDLSCs, there are plenty of studies investigating the immunomodulatory function of other DMSCs subpopulations. Researchers found that the exosomes derived from DPSCs had immunosuppressive properties, even stronger than the exosomes derived from BMSCs (Ji et al., 2019). DPSCs are of low immunogenicity, could inhibit the proliferation of lymphocytes via TGF-β1, and regulate the production of cytokines *in vitro* (Ding et al., 2015). In the context of allogeneic stem cell-based therapies, DPSCs inhibited peripheral blood mononuclear cell proliferation in a non-cell contact manner (Wada et al., 2009).

In recent years, GMSCs gradually attract more attention for tissue engineering applications due to their easy accessibility and excellent immunomodulatory property (Fawzy El-Sayed and Dörfer, 2016). Analogous to PDLSCs, GMSCs were reported to be able to polarize macrophages into the M2 phenotype *via* enhanced secretion of IL-6 and granulocyte–macrophage colony-stimulating factor (GM-CSF) (Zhang et al., 2010). In addition, GMSCs could significantly inhibit the maturation and differentiation of DCs derived from peripheral blood monocytes and could suppress a variety of inflammatory cytokines as well (Su et al., 2011). Most recently, a comprehensive study conducted through scRNA-seq also validated that some sub-clusters of GMSCs showed a potential role in immunomodulation. However, these clusters appeared to display pro-inflammatory nature, with enrichment for gene ontology (GO) terms such as T-cell activation, cytokine-mediated signaling pathway, and interferon-gamma (IFN-γ) signaling (Caetano et al., 2021). Given the characteristics of the oral cavity, a complex ecosystem containing numerous microorganisms with different pathogenic potentials, it would be interesting to examine whether DMSCs possess direct antimicrobial activity like BMSCs.

## Application

### Reconstruction of Bone Defects

Considering the prominent osteogenic differentiation ability, DMSCs are widely used to repair bone defects in combination with different types of scaffolds. Recently, a research group evaluated the efficacy of bone regeneration by using pre-differentiated GMSCs together with a self-assembling hydrogel scaffold to repair the critical size maxillary alveolar bone defect in a rat model. At 4 and 8 weeks after surgery, they observed a significant enhancement in bone regeneration compared with the control (Kandalam et al., 2020). DFSCs have also exhibited the properties of promoting new bone formation combined with treated dentin matrix (TDM) particles, a kind of scaffolding material, in a rat calvarial bone defect model (Yang H. et al., 2019).

Together with DMSCs and scaffolds, some chemical compounds were administered simultaneously to assess their pro-osteogenic functions in bone tissue engineering. As was mentioned earlier in this review, curcumin has been proven to activate osteogenic differentiation of DMSCs *in vitro* (Xiong et al., 2020). Researchers prepared guided bone regeneration (GBR) membrane with curcumin and aspirin addition. By utilizing this novel GBR membrane and DPSCs, bone regeneration was promoted, and meanwhile, antimicrobial effects were observed (Ghavimi et al., 2020). A similar result was found by another group that aspirin, in tetra-PEG hydrogel-based aspirin-sustained release system, could enhance the osteogenic capacity of PDLSCs (Zhang et al., 2019).

As a novel therapeutic option, tissue engineering strategies and their optimization attract attention and investigation continuously (Bajestan et al., 2017). Recently, researchers for the first time used deciduous DPSCs associated with a hydroxyapatite-collagen sponge to treat alveolar defects of cleft lip and palate patients and achieved satisfactory bone healing results (Tanikawa et al., 2020). In the dental research field, researchers also give attention to the regeneration of periodontal defects with DMSCs. GMSCs in combination with β-calcium triphosphate (β-TCP) scaffold could successfully reduce the vertical pocket depth and improve the cellularity of regenerated periodontal tissues (Abdal-Wahab et al., 2020). However, another similar study by using PDLSCs and a xenogeneic bone substitute (XBS) demonstrated no statistically significant differences concerning healing and re-ossification whether PDLSCs were present or not (Sánchez et al., 2020), which suggests that we should interpret experimental results with extra caution when elucidating the precise functions and effects of DMSCs.

### Tooth Regeneration

As is aforementioned, DMSCs are clonogenic cells and possess multiple differentiation potentials, which are conceived as a suitable cell source for dental tissue engineering and regeneration. Indeed, DMSCs are of profound significance in the regeneration of enamel, dentin, and pulp tissues. Transplantation of DPSCs *in situ* with granulocyte-colony-stimulating factor (G-CSF) has already been proven to result in the successful regeneration of vascularized pulp tissue in canine teeth (Iohara et al., 2013). A more pronounced volume of dental pulp-like tissue with a higher capillary density can be derived through a similar process *in vitro* with bFGF and G-CSF (Takeuchi et al., 2015). Researchers have also reported the successful regeneration of vascularized pulp-like tissue in an ectopic root transplantation model (Johnston et al., 2008). The shape of root canals and the size of the apical foreman are the key elements to focus on when judging the results. DPSCs derived from inflammatory dental pulp tissue have similar biological characteristics compared with those from normal dental pulp and could mediate pulp and dentin regeneration as well (Ling et al., 2020). The development of organoid or spheroid techniques for functional tooth regeneration has progressed rapidly. Several studies have demonstrated that organoids or spheroids of dental pulp cells were beneficial in the expression of VEGF (Hsieh et al., 2018), the capacity of angiogenesis (Janjić et al., 2018), and the formation of vascular dental pulp-like tissue (Dissanayaka et al., 2014). Hence, organoids or spheroids might constitute a practical and promising option for future clinical regenerative therapy (Oshima et al., 2017).

Different biocompatible scaffolds have been widely explored. Collagen can help SHEDs shape themselves into capillary-like microvessels (Sakai et al., 2010). Platelet-rich fibrin and chitosan both have been shown to contribute to dental pulp tissue engineering (Huangqin and Mingwen, 2007; Zhang et al., 2013). Polylactic acid (PLA) and polyglycolic acid (PGA) scaffolds are synthetic polymers that have been demonstrated to support cell proliferation and angiogenesis when attaining sufficient cell density (Woo et al., 2009). Another artificial biomaterial, polyhydroxybutyrate (PHB)/chitosan/nano-bioglass (nBG) nanofiber scaffold, has been revealed to promote SHEDs to differentiate into odontoblast-like cells and thereafter dentin formation (Khoroushi et al., 2018). Among all the biomaterials, fibrin appeared to be the most promising scaffold materials for dental pulp tissue engineering (Galler et al., 2018). Its advantages include easy handling, reasonable price, high cytocompatibility, and facilitation of pulp-like tissue formation.

For dentin mineralization and regeneration, the underlying mechanism is complicated and not clear yet. Therefore, most related studies concentrated on describing histological outcomes without digging into mechanisms. A study has proved that DPSCs organized in spheres are suitable for dental tissue engineering, which is capable of differentiating into odontoblast-like cells, inducing mineral formation, and display the possibility of “dentinal filling” of the root canal (Neunzehn et al., 2017). In addition, the microbiota appears to have an impact on dentinogenesis (Su et al., 2020) as well as some fabricated materials. To illustrate, nano-monetite hydrosol (nMH) contributed to dentin remineralization and acid resistance, but the interaction between nMH and DPSCs has not been discussed (Tan et al., 2020). Human β-defensin 4 (HBD4) controlled the intensity of dental pulp inflammation in a rat model of reversible pulpitis and induced the formation of restorative dentin (Zhai et al., 2020). For future research, studies should be conducted to reveal the interaction of DMSCs and scaffolds, and, more importantly, the underlying mechanisms in the context of tooth regeneration *in vivo*.

### Treatment of Periodontitis

Case definition of periodontal disease in epidemiological studies is a challenge (Peres et al., 2019), but is generally manifested with the loss of periodontal supporting tissues, including periodontal ligament, cementum, and alveolar bone. The ultimate goal of periodontal therapy is to achieve the regeneration of periodontal tissues. All regenerative therapies have shown that the formation of new cementum is a common critical step because the deposition of cementum precedes the attachment of new periodontal ligament fibers to the root surface (Han et al., 2014; Nuñez et al., 2019). So far, DMSC-based therapy has become one of the most promising approaches to periodontal tissue regeneration.

The periodontal ligament is the major component of the periodontium connecting the root surface of the tooth and alveolar bone. It is comprised of heterogeneous cell populations, which include fibroblasts, undifferentiated MSCs, and epithelial cells, such as the epithelial cell rests of Malassez. PDLSCs can be isolated from adults’ periodontal ligament tissues with the ability to generate alveolar bone, periodontal ligament, and cementum, as well as the potential of self-renewal (Martínez et al., 2011; Tomokiyo et al., 2018). Previous studies suggested that TGF-β1 played a crucial role in the fibroblastic differentiation process of PDLSCs (Fujii et al., 2010). Moreover, VEGF can stimulate the osteogenic differentiation of PDLSCs *in vitro* and induce the mineralization of bone structure (Lee et al., 2012).

Gingival mesenchymal stem cells have been proven to have multi-directional differentiation ability. One group demonstrated that transplanted GMSCs could accurately reach the periodontal injury sites and facilitate periodontal tissue regeneration by tail vein injection (Sun et al., 2019). A similar phenomenon was also observed in damaged periodontal tissues in a dog model (Yu et al., 2013). Another group used miniature pigs to establish an experimental periodontitis model and delivered SCAPs by local injection for treatment purposes, which verified the superior regenerative effect of periodontal tissue in the SCAPs treatment group (Li et al., 2018). Although the two groups of researchers both have proven that DMSCs effectively promote the regeneration of periodontal tissues, they applied different delivery approaches to injecting DMSCs. Plus, the mechanisms of how GMSCs or SCAPs promote periodontal tissue regeneration are not uncovered yet. Studies should put sufficient effort into clarifying the functions and mechanisms of DMSCs during periodontal tissue regeneration. As for DPSCs, the therapeutic effects are vaguer, though DPSCs indeed have the potential to differentiate into adipogenic, osteogenic, chondrogenic, and neural cells. Some studies have concluded that DPSCs successfully accomplish periodontal tissue repair and regeneration (Liu et al., 2011); however, some studies suggested that DPSCs hardly ever repaired the defects of periodontal tissue (Park et al., 2011). On the contrary, PDLSCs might be a more favorable candidate for clinical application than DFSCs and DPSCs (Park et al., 2011). Different conclusions indicate that the specific role of each subpopulation of DMSCs in periodontal tissue regeneration is still waiting for more detailed and in-depth exploration.

### Repair of Cartilage Injury

The chondrogenic potential of DMSCs is usually investigated in the presence of biological materials. Platelet-rich plasma (PRP) is a promising biomaterial that can be used as tissue engineering scaffolds (Pietrzak and Eppley, 2005). TGF-β1, as a conventional additive for chondrogenic differentiation, combined with a 10% PRP conditioned medium significantly potentiated chondrogenesis of DPSCs *in vitro* (Salkın et al., 2021). Recently, it has also been reported that nanoscale thermosensitive hydrogel scaffolds carrying human DPSCs could promote cartilage formation both *in vitro* and *in vivo* (Talaat et al., 2020). Moreover, the store condition of MSCs matters in tissue engineering. Researchers found that long-term cryopreservation in 95% FBS with 5% DMSO could maintain the chondrogenic capacity as well as the colony-forming ability of MSCs (Fujisawa et al., 2019).

Furthermore, there are some applications of DMSCs related to cartilage repair in osteoarthritis (OA). OA is a degenerative and inflammatory joint disorder, which takes place when the cartilage between the ends of bones gradually deteriorates. Recently, researchers revealed the protective effects of SHED-derived conditioned medium on OA chondrocytes. They found that OA chondrocytes exhibited an enhanced ability of anti-inflammation as well as a higher level of ACAN and COL2 when treated with SHED-derived conditioned medium (Muhammad et al., 2020). In addition, DPSC-derived conditioned medium could increase the survival and proliferation of immature murine articular chondrocytes *in vitro*; meanwhile, DPSCs directly underwent chondrogenesis as well (Lo Monaco et al., 2020).

### Wound Healing

Wound healing is a complicated and meticulously organized process that restores the integrity and function of the damaged tissue. The process of wound healing involves the interaction of different cell types, including endothelial cells, platelets, inflammatory cells, and fibroblasts, etc. These cells secrete VEGF, bFGF, PDGF, TGF-β, placental growth factor (PlGF), and tissue inhibitor of metalloproteinases 2 (TIMP-2) as well as other factors to regulate the healing process and modulate the extracellular matrix components. Researchers revealed that bFGF-treated SHEDs could significantly increase collagen fibril coverage in wounds and, thus, promote the healing process (Nishino et al., 2011). Moreover, wound healing facilitated by DMSCs is not merely confined to fibroblastic proliferation and/or collagen accumulation. A recent study showed that DPSC treatment stimulated re-epithelialization and ameliorated collagen deposition in healing wounds; in dystrophic mice, DPSC treatment resulted in reduced fibrosis and collagen content, and infiltration of higher numbers of proangiogenic M2-like macrophages, which causes more satisfactory healing status (Martínez-Sarrà et al., 2017). Preclinical studies suggest that high-intensity laser therapy and photobiomodulation therapy may collaborate with DMSCs to soften cells, reorganize cytoskeleton, and improve the efficacy of wound healing (Daigo et al., 2020; Malthiery et al., 2020).

### Repair or Regeneration of Neural Tissue

Dental-derived mesenchymal stem cells can be used as an ideal source for neural tissue repair and regeneration. All types of DMSCs can express BDNF, GDNF, nerve growth factor (NGF), NT-3, angiopoietin 1 (ANGPT1), and VEGF, participating in neural repair by exerting paracrine effects (Kolar et al., 2017; Ullah et al., 2017). Moreover, the expression levels of the abovementioned markers are highly increased following neural induction (Yamamoto et al., 2016). DMSCs promote axon regeneration through their neurotrophic functions, which could enhance the growth rate of Schwann cells and promote angiogenesis (Yamamoto et al., 2016; Ullah et al., 2017). Several studies have reported that BDNF plays an important role in neurite outgrowth and axonal targeting, which is regulated by DMSCs (De Almeida et al., 2014; Kolar et al., 2017). Moreover, transplantation of GMSCs positively modulated peripheral nerve regeneration and remyelination of Schwann cells, by the antagonistic myelination regulators (Zhang et al., 2016).

Dental-derived mesenchymal stem cells differentiate into neuron-like cells and replace the damaged cells. For instance, DPSC-derived oligodendrocytes significantly increased the myelination of peripheral nerves *in vivo*, suggesting the possibility of their use in demyelinating diseases (Askari et al., 2015). Moreover, DPSCs can differentiate into retinal ganglion cells, which also suggested the potential for the treatment of neurodegenerative diseases such as glaucoma (Bray et al., 2014; Roozafzoon et al., 2015). Additionally, DPSCs and SHEDs co-cultured with auditory brainstem slice (ABS) possess the ability to differentiate into spiral ganglion neuron-like cells, revealing the possibility of being a therapeutic approach for sensorineural hearing loss patients (Gonmanee et al., 2020). Meanwhile, the therapeutic effects of DMSCs have been tested in ischemic vascular diseases and observed neuroregeneration (Zhang Q. et al., 2018). In the rat model of middle cerebral artery occlusion (MCAO), DPSCs were administered intravenously and were found to migrate into ischemic areas and function as neuron-like cells, and consequently reducing infarct range.

Dental-derived mesenchymal stem cells can improve neural repair while suppressing neural cell apoptosis. Spinal cord injury (SCI) often causes a broad range of dysfunction, due to the loss of neurons and glia as well as the limited axonal regeneration after SCI. Transplanting human DPSCs into the completely transected adult rat spinal cord led to the remarkable recovery of hind limb locomotor functions (Sakai et al., 2012). DPSCs displayed three kinds of neuroregenerative properties in SCI recovery. First, the apoptosis of neurons, astrocytes, and oligodendrocytes, induced by SCI, was inhibited, which improved the preservation of neuronal filaments and myelin sheaths. Second, multiple axon growth inhibitors were directly inhibited by DPSCs via paracrine mechanisms. Third, DPSCs replaced lost cells through differentiating into mature oligodendrocytes. Another group showed that SHEDs improved motor function after SCI, by reducing TNF-α expression, the cystic cavity, and the glial scar (Nicola et al., 2016).

Furthermore, DMSCs have the potential to restore neurons in neurodegenerative diseases of the central nervous system (CNS), such as Alzheimer’s disease (AD) (Mita et al., 2015; Wang F. et al., 2017) and Parkinson’s disease (PD) (Chen et al., 2020b). DPSCs could inhibit the phosphorylation of tau protein and promote the proliferation of neural stem cells in an animal model of AD (Wang F. et al., 2017). The exosomes secreted by DMSCs are able to cross blood–brain barriers (Jiang and Gao, 2017; Stanko et al., 2018). Some exosomes can reduce cytotoxicity and apoptosis triggered by amyloid-beta (Aβ) peptide (Ahmed et al., 2016). Thus, when translated into clinical application, the administration of DMSC-conditioned medium or DMSC-derived exosomes might be more practicable and safer. DMSCs coupled with three-dimensional frames had an enhanced ability to facilitate facial nerve recovery and regeneration after injury (Sasaki et al., 2014; Zhang Q. et al., 2018). Local delivery of GMSC-derived exosomes could markedly improve sciatic nerve regeneration and functional recovery (Mao et al., 2019). Additionally, DPSCs have superior migration potential toward the neurodegenerative milieu compared with BMSCs (Senthilkumar et al., 2020). Collectively, these studies indicate that DMSC-derived exosomes and/or DMSC-conditioned medium can be effective in neuroregeneration, through regulating anti-inflammatory, neurogenic, anti-apoptotic, angiogenic, and osteogenic mediators.

### Treatment of Cardiovascular Diseases

Stroke is one of the most prevalent cardiovascular diseases worldwide. Recently, the advances in stem cell-based therapies to treat stroke have been reviewed (Suda et al., 2020). In a clinical trial, autologous BMSCs transplantation by intravenous infusion has been proven to be a feasible and safe therapy that could improve functional recovery in patients with severe cerebral infarcts (Bang et al., 2005). As for DMSCs, systemic delivery of DPSCs after reperfusion could reduce ischemic brain damage and improve functional recovery in a rat model (Nito et al., 2018). DPSC transplantation significantly reduced microglial activation and the expression of pro-inflammatory cytokines after reperfusion (Nito et al., 2018). Therefore, some researchers indicated that DPSCs might be a better choice of stem cell-based therapy for ischemic stroke than BMSCs (Song et al., 2017). Considering the neural differentiation potential of DPSCs, some studies delineated the transcriptional profile of DPSCs migrated to ischemic areas, which expressed neural markers, such as β-III-tubulin, doublecortin, nestin, and neurofilament (Zhang X. et al., 2018; Lan et al., 2019; Chen et al., 2020a). More randomized, double-blind, placebo-controlled, multicenter clinical trials are required to carefully evaluate the safety and efficacy of DMSCs in the treatment of ischemic stroke patients.

Ischemic heart diseases may cause irreversible damage to heart tissues, such as myocardial infarction (Song et al., 2017). In recent years, studies have demonstrated that DMSCs promoted the survival of cardiomyocytes in response to hypoxia and serum deprivation (Song et al., 2017). Furthermore, SHED-derived conditioned medium attenuated the LPS-induced expression of pro-inflammatory mediators. In brief, the cellular mechanism was the suppression of apoptosis and inflammatory reactions of cardiac myocytes (Yamaguchi et al., 2015).

### Treatment of Autoimmune Diseases

Systemic lupus erythematosus (SLE) is a prototypic autoimmune disease, with abnormalities in the innate immune system as well as the adaptive immune system. In a recent study, GMSCs were found to limit the development of autoantibodies and proteinuria, which decreased the frequency of plasma B cells and lupus nephritis histopathological score by directly inhibiting B-cell activation, proliferation, and differentiation (Dang et al., 2020).

Multiple sclerosis (MS) is a long-lasting progressive autoimmune neurological disorder of the CNS. Immune cell invasion, axonal injury, and myelin sheath deformation are the common hallmarks of MS, which eventually cause neurological disability (Frohman et al., 2006). The immunosuppressive role of PDLSC-derived conditioned medium and exosomes might partly attribute to the presence of soluble immunomodulatory factors, NALP3 inflammasome inactivation, and, thus, resulting in the alleviation of MS (Soundara Rajan et al., 2017). DMSCs can also modulate inflammatory, oxidative stress, and apoptotic pathways in an experimental model of MS (Giacoppo et al., 2017).

### Treatment of Liver Diseases

Liver cirrhosis, characterized by extensive fibrosis and the replacement of normal liver architecture into abnormal nodules due to diffused degeneration and death of hepatocytes, is the terminal stage of a variety of chronic liver diseases (Tsochatzis et al., 2008). As was aforementioned, DMSCs have the potential of differentiating into hepatocyte-like cells. For therapeutic applications, it has been reported that SHED-derived hepatocyte-like cells were positive for all examined hepatic markers (Yokoyama et al., 2019). Moreover, SHED-derived hepatocyte transplantation eliminated liver fibrosis and restored liver structure in rats (Yokoyama et al., 2019). Unfortunately, the number of *in vivo* experiments and preclinical studies remains very limited.

### Treatment of Urinary Diseases

Bladder augmentation or replacement is required in a variety of urological disorders, which may be caused by cancer, spinal cord injury, etc. SMC regeneration is an essential step in tissue engineering of the urinary bladder (Song et al., 2016). In a rat model of stress urinary incontinence with pudendal nerve transected, human DPSCs were engrafted in the external urethral sphincter, the thickness of which was mostly recovered 4 weeks later. DPSCs committed toward myogenic lineage *in vivo* promoted the formation of blood vessels and resulted in an appreciable recovery of urinary continence (Zordani et al., 2019). However, to generate functional, contractile, and mature SMCs through DMSCs, still there is a long way to go before making it a clinically available approach.

## Conclusion

Dental-derived mesenchymal stem cells are comprised of several distinct subpopulations, including, but not limited to, DPSCs, SHEDs, PDLSCs, DFSCs, SCAPs, and GMSCs, with multi-directional differentiation potentials as well as immunomodulatory functions. Besides, each subpopulation of DMSCs is not equivalent in terms of their biological properties. Hereby, to the best of our knowledge, we have thoroughly reviewed the multipotency of DMSCs, including odontogenic, cementogenic, osteogenic, chondrogenic, myogenic, neurogenic, angiogenic, hepatogenic, and adipogenic differentiation. However, we have noticed that numerous studies concerning the differentiation of DMSCs are conducted *in vitro* in the presence of different induction culture media, regardless of DMSCs’ specific origin, natural environment, and real behaviors *in vivo*.

Despite the possible discrepancy between the *in vitro* and *in vivo* differentiation potentials, we still believe that DMSCs are *bona fide* multipotent stem cells and feasible for a variety of clinical applications, such as soft and hard tissue engineering, tooth regeneration, and treatment of degenerative diseases. To this end, more experiments are a prerequisite for clinical translation. Not only more basic research is needed to unravel the regulatory mechanisms but also more preclinical and clinical studies are required to optimize and ensure the efficacy of DMSC-based therapy.

## Author Contributions

YM, BL, and ZZ contributed to the design, review, and proofreading of the manuscript. BL, TO, and YC contributed to the material collections and analysis. YC and YM contributed to the design of the figures. All authors agreed with the submission of the final version of the manuscript.

## Conflict of Interest

The authors declare that the research was conducted in the absence of any commercial or financial relationships that could be construed as a potential conflict of interest.
